# Isolation, characterization, and application of the novel polyvalent bacteriophage vB_EcoM_XAM237 against pathogenic *Escherichia coli*

**DOI:** 10.1186/s13567-025-01514-y

**Published:** 2025-04-24

**Authors:** Jiyun Chai, Hongfei Sun, Stefan Schwarz, Yuxuan Huang, Shuangyu Xie, Qiu Xu, Longhua Lin, Caiping Ma, Jie Hou, Yao Zhu, Wanjiang Zhang

**Affiliations:** 1 State Key Laboratory for Animal Disease Control and Prevention, Harbin Veterinary Research Institute, Chinese Academy of Agricultural Sciences, Harbin, 150069 China; 2 Institute of Microbiology and Epizootics, Centre for Infection Medicine, School of Veterinary Medicine, Freie Universität Berlin, 14163 Berlin, Germany; 3 Veterinary Centre for Resistance Research (TZR), School of Veterinary Medicine, Freie Universität Berlin, 14163 Berlin, Germany

**Keywords:** Bacteriophage, pathogenic *Escherichia coli*, biological characteristics, genome analysis, phage therapy

## Abstract

A novel polyvalent broad-spectrum phage, vB_EcoM_XAM237 (XAM237), was isolated from pig farm sewage. It can simultaneously lyse multiple strains of pathogenic *Escherichia coli* (*E. coli*), demonstrating a broad host range. When the enteropathogenic *E. coli* (EPEC) strain E711 was used as the host bacterium, the phage XAM237 exhibited a short latent period, high stability at different temperatures and pH values and good tolerance to chloroform. Moreover, phage XAM237 can efficiently adsorb and lyse host bacteria in vitro. Whole-genome sequencing revealed that XAM237 is a double-stranded DNA (dsDNA) phage consisting of 170 541 bp with a G + C content of 35%. Phylogenetic analysis confirmed that XAM237 belongs to the family *Straboviridae*, genus *Tequatrovirus*. In addition, the genome of XAM237 did not contain genes related to lysogenicity, virulence or antimicrobial resistance. The effects of phage XAM237 in treating EPEC infections in vivo were evaluated in a mouse model. Phage XAM237 was able to reduce the number of colonized aEPEC strain E711 in the small intestine, liver, spleen, and kidney. This study suggested that phage XAM237 may be a promising candidate biologic agent for controlling pathogenic *E. coli* infections.

## Introduction

Pathogenic *Escherichia coli* (*E. coli*) is a leading cause of diarrheal disease in newborn and early-weaned piglets, causing substantial economic losses through increased morbidity, mortality, and treatment costs, as well as reduced growth rates in affected piglets [1]. There are four different pathotypes of this bacterium that can cause diseases in swine: enterotoxigenic *E. coli* (ETEC), Shiga toxin-producing *E. coli* (STEC), enteropathogenic *E. coli* (EPEC), which comprises typical EPEC (tEPEC) and atypical EPEC (aEPEC), and extraintestinal pathogenic *E. coli* (ExPEC). Among these, the most relevant pathotype in a colibacillosis scenario is ETEC, which includes five common antigenically different types found in pigs: F4 (K88), F5 (K99), F6 (987P), F18, and F41 [2]. To control infections, antimicrobial agents are widely used in breeding farms to treat infections. However, *E. coli* has been reported to be resistant to multiple antimicrobial agents, complicating treatment strategies [3]. The prevention and control of bacterial infections urgently require the development of alternative antimicrobial products.

Bacteriophages (phages), the most abundant biological entities on Earth, are viruses that specifically infect and kill bacteria, offering a promising alternative to conventional antimicrobial agents [4, 5]. Owing to their unique advantages, such as high specificity for target bacteria, reduced likelihood of resistance development, and ability to self-replicate at the site of infection, phages are gaining increasing attention as potential solutions to the antimicrobial resistance crisis [6, 7]. Despite these benefits, the host range of most characterized phages thus far is typically restricted to a single bacterial species, with few capable of infecting multiple species. The use of bacteriophage cocktails, or phage cocktails, offers a broad-spectrum approach to combat bacterial infections. However, the multicomponent nature of these cocktails poses challenges, as simultaneous exposure to various phages could impose selective pressures that foster the emergence of resistance mechanisms in bacteria [8]. The use of a single phage with a broader host spectrum can reduce resistance development, and the relative screening and preparation process is faster and less costly. The use of a single, broader-spectrum phage may mitigate this risk, offering a more streamlined screening and preparation process that is both time-efficient and cost-effective. It is imperative to gain a comprehensive understanding of phage biology and genetic profiles to guarantee both their efficacy and safety for therapeutic applications. A suitable phage candidate for effective biocontrol should be a lytic phage with a broad host range against a variety of bacterial strains and should not carry virulence, resistance or pathogenicity genes in the genome. To date, the reported phages in the database have only been the tip of the iceberg, and most of them belong to well-studied families, such as *Myoviridae* and *Siphoviridae*, which are characterized by their distinct tail structures [9, 10]. However, the vast majority of phages, including those from less explored families or with unique properties, remain to be discovered and characterized.

In this study, a novel polyvalent bacteriophage, XAM237, was isolated from farm sewage and characterized. Its biological properties were evaluated, and its genome sequence was obtained and analysed in comparison with those of related phages. Phage XAM237 has a wide host range and can lyse multiple pathotypes among pathogenic *E. coli* strains. In addition, phage XAM237 showed a good protective effect in a mouse model challenged with pathogenic *E. coli*. These data provide valuable information for assessing the potential of phage XAM237 as a biocontrol agent against pathogenic bacteria.

## Materials and methods

### Bacterial strains, phage isolation and animals

The strains used in this study are listed in Table 1. Among these strains, reference strains were obtained from the China Center of Industrial Culture Collection (CICC), the China Veterinary Culture Collection Center (CVCC) and the National Center for Medical Culture Collections (CMCC), whereas the others were isolated from breeding farms in northern China by our laboratory. The typical enteropathogenic *E. coli* (aEPEC) strain E711 was selected as the host strain for screening and amplifying the phage. All strains were preserved in 25% glycerol (v/v) at −80 °C and revived in Luria–Bertani (LB) medium at 37 °C overnight.

**Table 1:** **The host range of phage XAM237**

Bacterial strains	Strain nos	Source	Plaque^a^	Spot testing^b^
Atypical enteropathogenic *E. coli*	E711	Pig	+	+ +
Enterotoxigenic *E. coli* K99	C83925	Pig	+	+
Enterotoxigenic *E. coli* F18	EC2024	Pig	+	+ +
Enterotoxigenic *E. coli* K88	EC2232	Pig	+	+ +
	EC1004	Pig	+	+ +
	EC1003	Pig	+	+ +
	EC2215	Pig	−	−
	EC2233	Pig	−	−
*Escherichia coli*	EC2001	Pig	+	+
	8–2-1	Pig	+	+ +
	6–3	Pig	+	+ +
	EC2005	Pig	−	−
	EC2016	Pig	−	−
	EC2035	Pig	−	−
	EC2041	Pig	−	−
	EC2009	Pig	−	−
	EC2010	Pig	−	−
	EC2011	Pig	−	−

^a^ + indicates plaque observed, − indicates no plaque observed

^b^ +  + indicates complete lysis, + indicates turbid lysis, and −indicates no lysis

Phage XAM237 was isolated and purified from pig farm sewage in the Inner Mongolia Autonomous Region via methods previously described by Zhang et al. [11]. The bacteriophage lysates were purified using a sterile disposable membrane filter (0.22 μm) and stored at 4 °C.

Female BALB/c mice, 5–6 weeks of age (15–16 g in weight), were purchased from Beijing Vital River Laboratory Animal Technology Co., Ltd. (Beijing, China).

### Phage purification and quality control

The purification method using CsCl density gradient centrifugation was adapted from the procedure described by Luong et al. [12]. Briefly, CsCl densities of 1.30 g/mL, 1.50 g/mL and 1.60 g/mL were carefully layered in 13.2 mL open-top thin wall ultraclear centrifuge tubes (Beckman, Australia) in 0.2 μm sterile filtered Tris‒sodium chloride buffer, pH 7.0 (10 mM Tris (pH 7.0) and 150 mM NaCl). After the weights of the samples in the centrifuge tubes were adjusted, approximately 4 mL of the crude phage suspension was added. The gradient was then centrifuged in a Beckman SW40 Ti rotor at 28 000 × *g* and 4 °C for 258 min (Beckman Coulter, XPN-100 Ultracentrifuge). Finally, the purified phage particles were extracted from the base of the visible white/gray band using a 26-gauge needle and a 3 mL syringe. The endotoxin concentration of the phage sample was subsequently measured using the ToxinSensorTM chromogenic LAL endotoxin assay kit from Beyotime Biotechnology, and the pH value was measured using a pH meter. The absorbance of the phage preparations at 280 nm was determined to measure protein levels. The phage titre was determined using the double-layer agar plate method, yielding a precise quantification of the phage concentration. These combined assessments ensured that the phage preparation was suitable for subsequent experimental use.

### Host range determination

Eighteen strains of *E. coli* (Table 1) were utilized to investigate the lytic capacity of phage XAM237 using the spot test method [13] to evaluate its potential to create lysis zones on lawn cultures of various bacterial strains. The purified phage XAM237 suspension (10 μL, 10^7^ PFUs/mL) was spotted directly onto the surface of a bacterial lawn culture plate and incubated at 37 °C overnight, after which the plates were observed for the presence of plaques.

### Transmission electron microscopy

A Hitachi H-7650 transmission electron microscope (TEM) was used to examine the purified phage suspension (10^9^ PFUs/mL) after incubation for 10 min on copper grids covered with carbon [14].

### TEM of phage‑infected bacteria

The effects of phage XAM237 on the ultrastructure of the EPEC strain E711 were visualized via TEM. Briefly, the EPEC strain E711 was infected with phage XAM237 at a multiplicity of infection (MOI) of 1 for 1 h. The samples were pelleted by centrifugation at 12 000 × *g* for 5 min at 4 °C and washed two times with 0.1 M PBS (pH 7.4). After fixation in 2.5% glutaraldehyde/PBS overnight at 4 °C, the samples were washed with PBS and then incubated with 1% (w/v) osmium tetroxide prepared in PBS for 2 h. The fixed samples were dehydrated in a series of ice-cold ethanol washes and then infiltrated with Jembed 812 resin. Thin sections were cut with an ultramicrotome and mounted on nickel grids, followed by staining with uranyl acetate and lead citrate. Micrographs were taken with a Hitachi H-7650 transmission electron microscope at an acceleration voltage of 100 kV.

### Phage titre and optimal multiplicity of infection

The phage titre was determined through the double-layer agar method after serial dilution of the phage stock solution and calculated via the following formula: PFUs/mL = number of plaques × dilution factor × 10. The MOI was expressed as the ratio of phage count to host bacterial count during infection. First, the logarithmic growth phase of the aEPEC strain E711 at a concentration of 10^8^ colony-forming units (CFUs)/mL [optical density at 600 nm (OD_600_ nm) = 0.60] was prepared on the basis of the correlation curve between the OD_600_ nm and CFUs. At MOIs of 0.001, 0.01, 0.1, 1, 10, 100, phage stock and bacteriophage stock were added to centrifuge tubes at 37 °C for 10 min. Subsequently, 10 mL of LB medium (containing 10 mM CaCl_2_) was added to the mixture, which was then incubated at 37 °C for 8 h. The precipitate was removed by centrifugation at 12 000 × *g* for 10 min. The precipitate was removed by centrifugation at 12 000 × *g* for 10 min, and the supernatant was filtered through a 0.22 μm syringe filter. The double-layer agar method was used to determine the phage titre, with the highest titre of infection being considered the optimal MOI [15].

### One-step growth curve

The following modifications were made to the one-step growth curve described earlier by Wei et al. [16]. First, the logarithmic growth phase of the aEPEC strain E711 at a concentration of 10^8^ colony-forming units (CFUs)/mL [OD_600_ nm = 0.60] was prepared on the basis of the correlation curve between the OD_600_ nm and CFUs. At an MOI of 0.01, the phage stock and bacteriophage stock were added to centrifuge tubes at 37 °C for 10 min, and no adsorbed phage particles were removed. The bacterial pellets were suspended in 10 mL of LB medium, followed by incubation at 37 °C. The double-layer agar plate method was used to plate samples for phage titre after they had been collected at different time points within 1 h. The burst size was calculated by subtracting the initial titre from the final titre and then dividing it by the initial titre [17].

### Thermal and pH stability assays

The thermal stability of phage XAM237 was tested at 4, 30, 37, 40, 50 and 60 °C in LB broth after incubation for 1 h. Then, double-layer plates were immediately constructed, and the phage titres after incubation at different temperatures were determined. In addition to the temperature settings above, phages stored at 4 °C were used as a control for measuring the titres.

To test the pH stability, phage XAM237 was suspended in LB broth at different pH values (adjusted from 1 to 12 with HCl and NaOH) and incubated for 1 h, followed by phage titre determination.

### Chloroform tolerance

The phage XAM237 suspension (100 μL) was mixed with 900 μL of chloroform and incubated for 1 h at 37 °C. The double-layer agar plate method was used to determine the resulting phage titre.

### Whole-genome sequencing analysis

The phage XAM237 suspension was first treated with RNase (3 μg/mL) and DNase I (0.1 U/μL) at 37 °C for 1 h to degrade the bacterial nucleic acids. The genome of phage XAM237 was extracted using the TIANamp Virus DNA/RNA Kit (Tiangen Biotech, Beijing, China), followed by genome sequencing using the Illumina NovaSeq platform. The de novo assembly of high-quality reads was performed using SPAdes v3.15.2.

Open reading frames (ORFs) were identified using the RAST server [18] and verified via the GeneMark server [19]. The putative functions of the encoded proteins were determined using BLASTp, and the similarity of phage genome sequences was assessed using BLASTn [16, 20]. Using tRNAscan-SE software (version 1.3.1), putative tRNA sequences were located. Antimicrobial resistance genes and virulence genes were detected using the CGE platform [21]. The genome map of phage XAM237 was constructed and visualized using the CGView server [22]. To determine the taxonomy of the phage, via the neighbour‒joining method in MEGA XI, an evolutionary tree of phage XAM237 and randomly selected previously published phages was constructed. The genomes of phage XAM237 were compared via Easyfig software [23].

### Adsorption properties

The aEPEC strain E711 was cultured to the exponential growth phase, and phage XAM237 was added at an MOI of 0.01. A total of 100 μL of the mixture of an EPEC strain E711 and phage XAM237 was taken at different time points within 30 min. The titres of the free phage XAM237 in the supernatant were determined by the double-layer method at each time point immediately after centrifugation. The adsorption percentages were calculated as [1—(phage titre of the supernatant after the cells were removed/phage titre of the control reaction mixture without bacterial cells)] × 100%.

### Assessment of phage lytic activity at different MOIs

The lytic activity of the phages against *E. coli* was examined in vitro. The aEPEC strain E711 was used as the host strain. The host strain cultured to early logarithmic growth was infected with phages at different multiplicities of infection (MOIs = 0, 0.01, 0.1, 1.0, 10) and incubated in a shaker at 180 rpm and 37 °C for 12 h. The OD_600_ nm was measured at 1 h intervals.

### Antibacterial activity of phage XAM237 in a mouse model challenged with the aEPEC strain E711

First, the phage XAM237 was prepared for use in a mouse model. Then, 10 mM CaCl₂ was added to 200 mL of LB medium in a conical flask. The medium was inoculated with the phage XAM237 at the optimal MOI, along with the EPEC strain E711. The mixture was incubated overnight at 37 °C. In accordance with Bourdin et al. [24], the mixture was centrifuged at 5000 × *g* for 10 min at 4 °C to remove bacterial debris. A 10 mL volume of the supernatant was layered on a 20% (wt/vol) sucrose cushion at the bottom of 13.2 mL open-top thin wall ultraclear centrifuge tubes (Beckman, Australia), and the tube was filled with PBS to the required volume, followed by ultracentrifugation at 35 000 × *g* for 40 min at 4 °C using a Beckman SW 41Ti rotor (Beckman Coulter, Optima XPN-100 Ultracentrifuge) to pellet the phage particles. After ultracentrifugation, the supernatant was carefully removed, leaving the phage pellet. The phage pellet was then resuspended in 1 mL of PBS to achieve a concentrated phage suspension. This concentrated phage suspension was subsequently subjected to further purification and quality control processes to ensure its suitability for therapeutic applications in mice.

To prepare the bacteria for mouse infection, the following procedure was employed: aEPEC strain E711, at a concentration of 10^7^ CFUs/mL, was centrifuged at 5000 × *g* for 10 min at 4 °C to pellet the bacteria. The pellet was then washed three times with phosphate-buffered saline (PBS) under the same centrifugation conditions (5000 × *g* for 10 min at 4 °C) after each resuspension. After the final wash, the pellet was resuspended in 100 μL of PBS, resulting in a final concentration of 10^7^ CFUs of the aEPEC strain E711 per 100 μL, which was then used for mouse infection.

To evaluate the protective efficacy of phage XAM237 in vivo. Thirty-five 5-week-old female BALB/c mice were randomly divided into five groups (a–e). Groups a to e were initially inoculated intraperitoneally with 10^7^ CFUs of the aEPEC strain E711. One hour after bacterial challenge, groups a to c were given a single intraperitoneal dose of phage XAM237, with volumes of 100 μL containing 10^6^, 10^7^, and 10^8^ PFUs, respectively. At this time, the mice began to exhibit diarrhea symptoms caused by aEPEC. Group d was treated with an intraperitoneal injection of 5 mg/kg gentamicin. Group e served as the negative control and received 100 μL of PBS instead of the phage. The animals were then monitored daily for a period of seven days to document their clinical symptoms and overall mortality rate. After the observation period, the mice were humanely euthanized, and their small intestines were collected. Tissue sections from these intestines were prepared and stained with haematoxylin and eosin (H&E) for microscopic analysis to identify any pathological changes.

To investigate the metabolic dynamics of bacteria and phages in the mice, 36 5-week-old female mice were randomly divided into three groups (f–h). Each group received an intraperitoneal inoculation of 10^7^ CFUs of the aEPEC strain E711. Group f was administered an intraperitoneal dose of 10^8^ PFU of the phage XAM237 in a volume of 100 μL, and group g was administered an intraperitoneal dose of 5 mg/kg gentamicin in a volume of 100 μL. The group that received a 100 μL injection of sterile PBS served as a negative control. The animals in groups f, g, and h were sacrificed at 1, 6, 12, and 18 h post-infection, with three mice euthanized at each time point to ensure data consistency. The bacterial and phage loads within the excised abdominal organs (spleen, liver, kidneys, and small intestine) of the mice were precisely determined through meticulous quantification. The detailed experimental procedures were as follows: abdominal organs were excised using sterile surgical instruments and immediately transferred into prechilled PBS to prevent sample contamination and bacterial degradation. The excised organs were weighed and placed into sterile Eppendorf tubes, followed by the addition of an appropriate volume of prechilled PBS (1 mL of PBS per 1 g of organ). After magnetic beads were added, the organs were homogenized using a high-throughput tissue homogenizer. The homogenized organ samples were then subjected to tenfold serial dilutions (ranging from 10^–1^ to 10^–6^). Aliquots of 10 µL from each dilution were spotted onto MacConkey agar plates containing 8 µg/mL colistin, which was used to screen the aEPEC strain E711. The plates were incubated at 37 °C for 12–24 h to observe CFUs, and the bacterial load was calculated as CFU/g of tissue. PCR was performed to detect the virulence genes of aEPEC, which specifically target the *eae*A gene, using the forward primer (5′-CTGAACGGCGATTACGCGAA-3′) and reverse primer (5′-CCAGACGATACGATCCAG-3′) [25]. An appropriate volume of the diluted homogenate was centrifuged at 5000 × *g* for 10 min at 4 °C to remove tissue and bacterial debris. The supernatant (100 µL) was used for plaque assays with the double-layer agar method to quantify phage plaques, and the phage load was calculated as PFU/g of tissue on the basis of the dilution factor and sample weight.

### Statistical analysis

For each time point, averages and standard deviations were computed across all experiments, which were run in triplicate. The results are expressed as the mean ± standard deviation (SD). The survival curves were compared using the Gehan-Breslow‒Wilcoxon test in the Kaplan‒Meier survival analysis. *P* values < 0.05 were considered statistically significant; *P < 0.05; **P < 0.01; ***P < 0.001 and ^ns^*P* > 0.05 for all analyses. The data were analysed with GraphPad Prism 7.0 (GraphPad Software, San Diego, CA, USA).

## Results

### Host range of phage XAM237

Phage XAM237 showed characteristics of a wide range across species, demonstrating lytic activity against different pathotypes of *E. coli*, including aEPEC, ETEC K88, K99 and F18 (Table 1).

### Morphological features of phage XAM237

**Figure 1:**
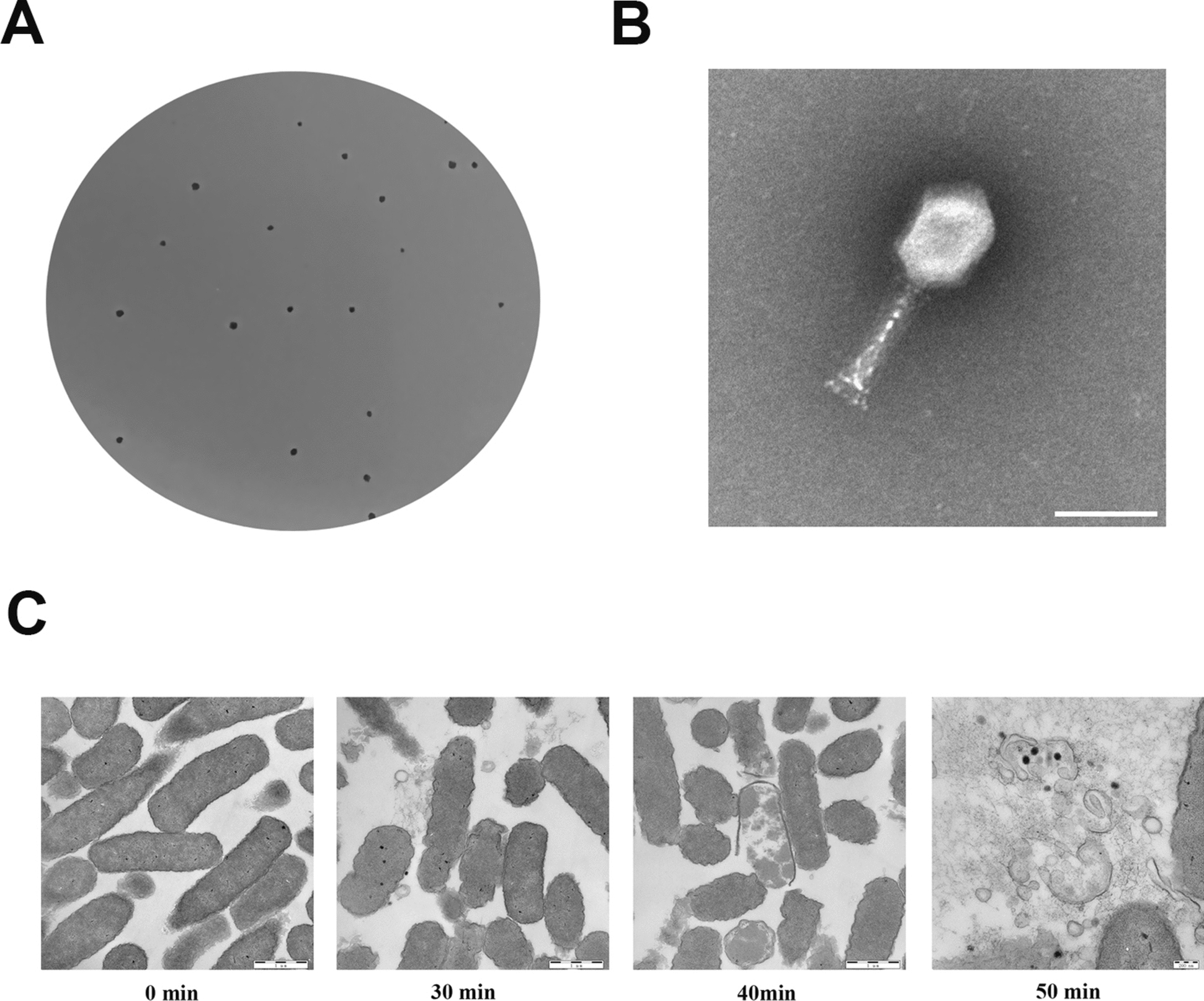
**Morphology of phage XAM237**. **A** Plaque morphology of phage XAM237. **B** TEM images of phage XAM237. The scale bars represent 200 nm. **C** TEM image of *E. coli* E711 infected with phage XAM237.

The phage XAM237 exhibited effective lytic properties against the aEPEC strain E711. XAM237 produced approximately 1 mm plaques after overnight culture on 0.6% agar plates (Figure 1A). On the basis of the TEM images (Figure 1B), phage XAM237 had a 70.62 nm diameter head and a 101.62 nm tail. Thus, phage XAM237 was classified as a member of the *Caudoviricetes* class on the basis of these structural characteristics. According to the novel universal system of bacteriophage naming, the suggested full name of phage XAM237 is vB_EcoM_XAM237 [26].

### Transmission electron microscopy of phage-infected aEPEC strain E711

TEM was used to examine the ultrastructural changes in phage-infected bacteria. Uninfected aEPEC strain, E711 cells displayed a regular morphology with an intact cell membrane (Figure 1C, 0 min). For phage XAM237-infected aEPEC strain E711 cells, the phage induced pits in the cell wall. The intracellular density changed, and the cell membrane disintegrated, leading to damage to E711 cells (Figure 1C, 30 min, 40 min) and, eventually, complete lysis of the cells (Figure 1C, 50 min). Large electron-dense granules were observed inside the cells.

### Biological characteristics of phage XAM237

**Figure 2:**
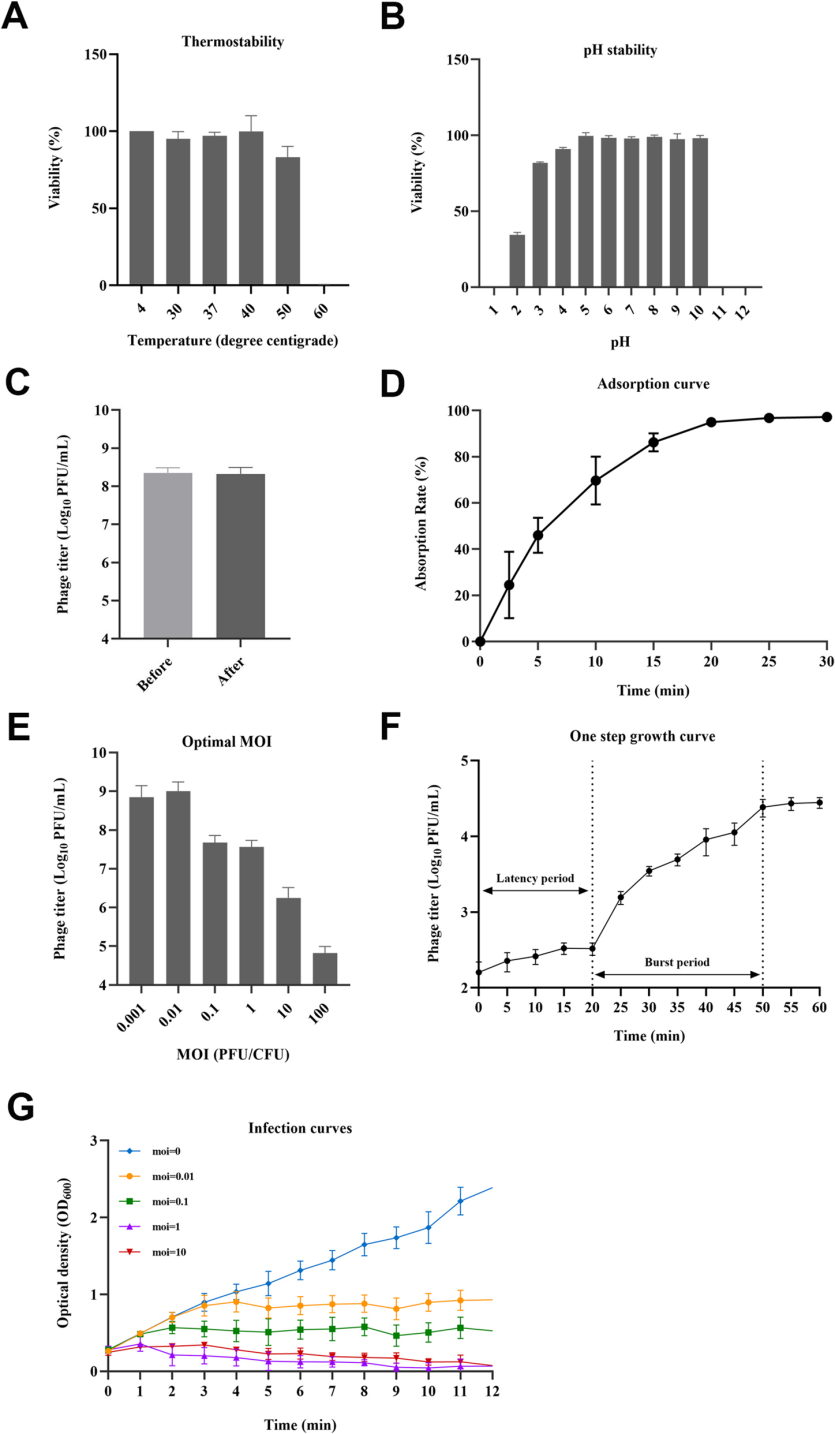
**Biological characteristics of phage XAM237**. **A** Thermostability of phage XAM237. **B** pH stability of phage XAM237. **C** Chloroform stability. **D** Adsorption curve of phage XAM237 to its host aEPEC E711. **E** Determination of the optimal MOI. **F** One-step growth curves of phage XAM237 in the presence of the host aEPEC E711. **G** Infection curve of phage XAM237.

**Figure 3:**
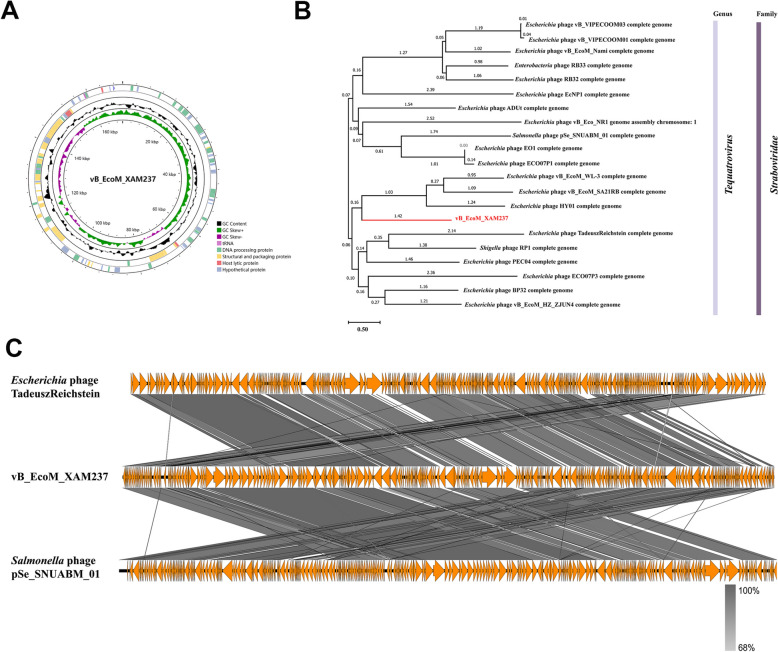
**Genomic analysis of phage XAM237.** **A** Circular genome map of phage XAM237. **B** Phylogenetic tree of phage XAM237 based on the whole phage genome. **C** Comparative linear genome of phage XAM237.

As shown by the thermostability and pH stability assays, the survival rate of phage XAM237 was greater than 80% in the ranges of 4–50 °C and pH 3.0–10.0, respectively (Figures 2A and B). When exposed to chloroform, phage XAM237 remained stable (*p* > 0.05) (Figure 2C). The adsorption of phage XAM237 to the host bacteria was rapid, taking up 5 min for 50% adsorption and 20 min for 95% adsorption (Figure 2D). Almost all the phages were adsorbed within 30 min. The adsorption curve indicated that phage XAM237 had a strong affinity for the host bacterial cells. The MOI of the phage was determined, as shown in Figure 3E. The phage titre reached the highest MOI of 0.01, indicating that the optimal MOI of phage XAM237 was 0.01. The one-step growth curve of phage XAM237 propagated on the host aEPEC strain E711 in LB medium revealed that the latent and rise periods were approximately 20 and 30 min (Figure 2F), respectively. The phage XAM237 had a burst size of 174 PFUs/infected cell. Phage XAM237 can effectively lyse host cells at various MOIs, even at MOIs as low as 0.01. At MOIs of 0.1, 1 and 10, the growth of the aEPEC strain E711 was gradually inhibited by the phage XAM237 within 1.0 h to 2.0 h. At an MOI of 0.01, the growth of the E711 strain began to be gradually suppressed after 3 h, with the suppression persisting throughout the study period. Notably, the OD_600_ value eventually approached 0 at MOIs of 1 and 10 (Figure 2G). Therefore, phage inhibition was more effective as the MOI increased. These findings indicated that the phage effectively inhibited the growth of the aEPEC strain E711.

### General genomic features

Phage XAM237 has a double-stranded DNA (dsDNA) genome consisting of 170 541 bp with a G + C content of 35% (Figure 3A). The presence of six tRNA genes was identified between 158 746 and 159 756 bp of the XAM237 genome. Compared with BLASTn, phage XAM237 displayed a high sequence identity of 96.41% (81% coverage) with the *Escherichia* phage TadeuszReichstein (GenBank accession no. MZ501106.1), followed by 96.63% identity (87% coverage) with the *Salmonella* phage pSe_SNUABM_01 (GenBank accession no. NC_054937.1) (Figure 3B). Phylogenetic analysis of the whole genome of the phage revealed that XAM237 belongs to the family *Straboviridae*, genus *Tequatrovirus,* and the diagram presented an independent branch for phage XAM237 (Figure 3C). Gene annotations revealed that phage XAM237 contained 272 predicted ORFs, of which 81 genes encoded phage-related proteins. Among them, 13 ORFs, including ORF062, ORF131, ORF133, ORF134, ORF182, ORF186, ORF199, ORF201, ORF206, ORF207, ORF209, ORF215, and ORF230, were annotated as structural proteins. Ten ORFs, including ORF009, ORF054, ORF070, ORF090, ORF091, ORF105, ORF111, ORF136, ORF210 and ORF213, were annotated to be related to the genome replication of bacteriophages. Notably, ORF130 and ORF255 encode holin and lysozyme R, which can lead to bacterial lysis. Genes related to lysogeny, virulence and antibiotic resistance were not detected in the phage genome.

#### Protective effects of phage XAM237 in a mouse model challenged with the aEPEC strain E711

The phage lysate used for therapy, after purification and quality control, had a pH of 7.5, a total protein concentration of 2.81 mg/mL, and an endotoxin concentration of 18.73 EU/mL. These parameters are crucial for ensuring the stability and safety of phage lysates for therapeutic applications.

**Figure 4:**
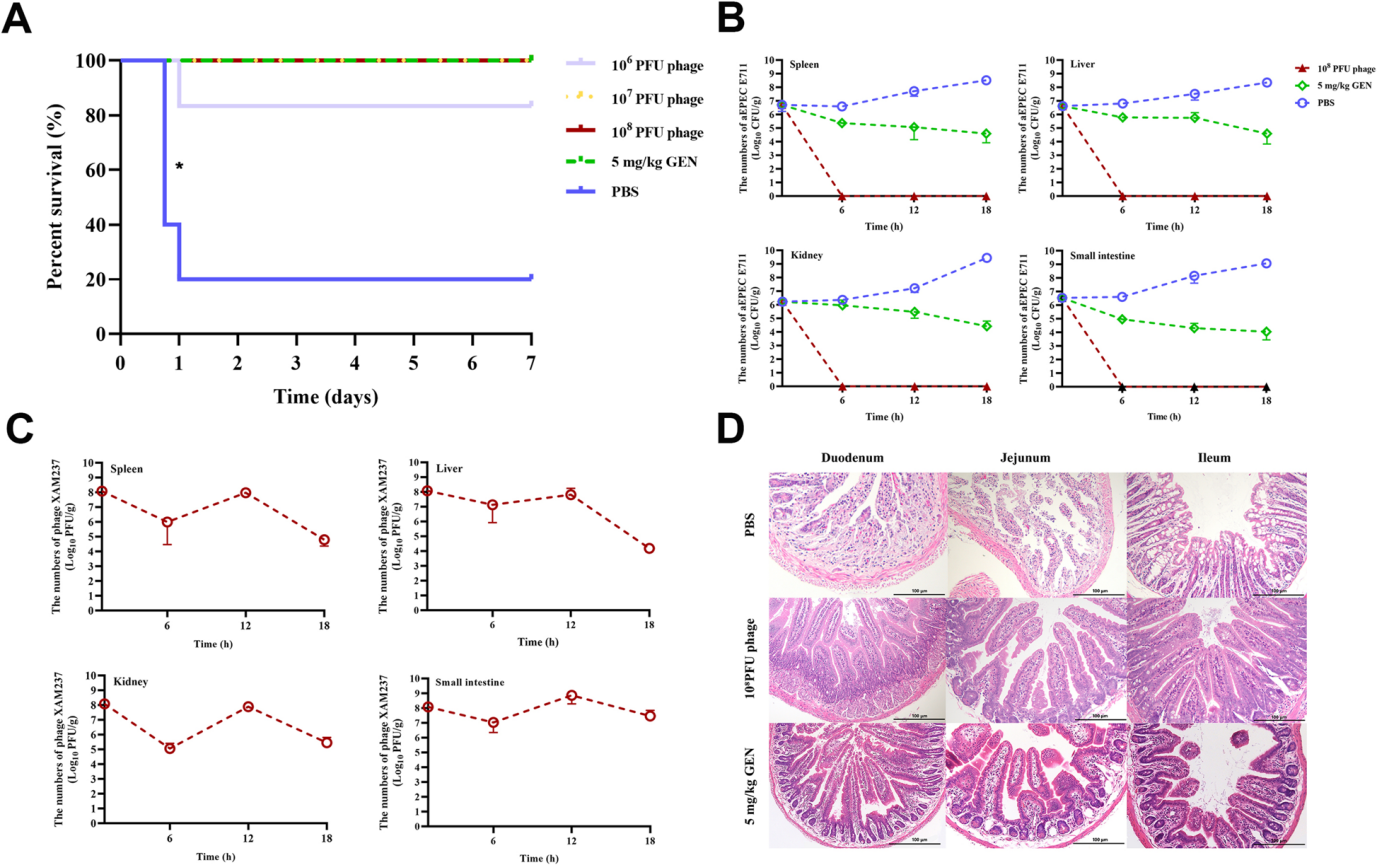
**Phage XAM237 rescued mice from aEPEC E711 infection**. **A** Survival rates. The mice were injected intraperitoneally with 10^7^ colony-forming units of the aEPEC E711 strain. One hour later, different phage doses were introduced intraperitoneally to treat the challenged mice. **B** Dynamic changes in bacteria in abdominal organs (spleen, liver, kidney, and small intestine) in different groups. **C** Dynamic changes in phage XAM237 in abdominal organs (spleen, liver, kidney, and small intestine) in different groups. **D** Pathology of the small intestine (duodenum, jejunum, ileum) in mice in different groups.

After challenge with the E711 strain, the survival rate of the mice in the untreated group was 20% within one day, indicating a high mortality rate. The phage-treated groups b (10^7^ PFUs) and c (10^8^ PFUs), as well as the gentamicin-treated group d, achieved a 100% survival rate, and the phage-treated group a (10^6^ PFUs) had a considerably lower survival rate of 80% (Figure 4A). The obvious protective effects of phage XAM237 were observed in bacteria-challenged mice (*p* < 0.05). In the untreated group h, the bacterial load in the small intestine, liver, spleen, and kidney progressively increased and reached 10^8^ to 10^9^ CFUs/g by 18 h. Notably, in the phage-treated group (10^8^ PFUs), a significant reduction in the bacterial burden in the small intestine, liver, spleen, and kidney was observed after five hours of treatment. Compared with that in the phage-treated mice, the bacterial load in the gentamicin-treated mice was significantly lower (Figure 4B). Furthermore, the levels of phage XAM237 in the small intestine, spleen, and kidney remained above 10^5^ PFU/g of tissue after treatment, indicating the stability of the phage within the body and its sustained antimicrobial activity (Figure 4C). Pathological observations of the small intestine revealed atrophy of the villi and edema in the lamina propria in the untreated group e, inflammatory infiltration in the lamina propria of the mucosa in the gentamicin-treated group d, and no lesions in the phage-treated groups c (Figure 4D). The results showed that XAM237 was able to provide adequate protection for all the mice without toxic side effects. Moreover, phage therapy is more effective than gentamicin treatment, which is commonly used for veterinary clinical treatment of colibacillosis.

In this study, we isolated and identified the phage XAM237, which is capable of infecting multiple strains of pathogenic *E. coli*. XAM237 has excellent lysis potential and biological safety. More importantly, phage XAM237 could clear bacteria in animals within a short time without side effects and has potential value in the treatment of bacterial diseases.

## Discussion

Phages, known for their remarkable abundance and genetic diversity, are widely acknowledged as the most successful biological entities on Earth [5], with an estimated 10^8^ virions per milliliter in natural water [27], 10^7^ to 10^10^ virions per gram of soil [28, 29], and 10^8^ to 10^10^ virions per milliliter in sewage [30]. Previous studies have identified sewage from wastewater treatment plants and animal waste as the primary sources of coliphages, with the foecal matter and gastrointestinal contents ranking as secondary sources [31]. In this study, we isolated the lytic phage XAM237 from farm sewage, which is capable of infecting multiple strains of pathogenic *E. coli*. The phages are devised by the International Committee on Taxonomy of Viruses (ICTV) according to the genome type, phage tail, and capsid/head morphologies. In accordance with the latest ICTV Taxonomy Release, the naming of bacterial viruses has been standardized according to the Linnaean nomenclature. Order *Caudovirales* and the families *Myoviridae*, *Podoviridae* and *Siphoviridae* were removed, and all underlying taxa were assigned directly to the class *Caudoviricetes* [32]*.*

The biological characteristics of phages are key factors in their therapeutic application [33]. Bacteriophages typically exhibit reduced stability in environments with extreme acidity, characterized by a pH below 3, primarily because acidic conditions can cause denaturation of their proteins. In general, bacteriophages are most stable and survive best in the pH range of 5–9, with an optimal pH of approximately 5–6 [34, 35]. In this study, phage XAM237 showed good stability over a wide pH range from 2–10. Temperature variations significantly influence the structure and function of biomolecules, including those of bacteriophages [36]. Many bacteriophages remain stable and retain their infectivity at low temperatures (approximately 0 °C). While some phages can withstand high temperatures, many are sensitive to extreme heat [34]. Phage XAM237 survived at temperatures ranging from 4 °C to 50 °C.

In phage therapy, the stability and infectivity of phages are paramount. Susceptibility to chloroform can influence the choice of phages used in therapeutic formulations, as chloroform-sensitive phages may not survive the processing conditions typically used in therapeutic preparations. Assessing chloroform tolerance provides critical insight into the presence of lipid components within the capsid or tail of bacteriophages, which can influence their suitability for therapy. Our findings revealed no significant difference in the titre between the untreated and chloroform-treated bacteriophage XAM237, suggesting that it lacks lipids in its structural components.

The efficacy of phage therapy is influenced by several critical parameters, including the phage adsorption rate, burst size, and latency period. The adsorption of phage XAM237 to the host bacteria was rapid, taking up 5 min for 50% adsorption and 20 min for 95% adsorption. The findings of the one-step curve revealed that the latent and rise periods were approximately 20 and 30 min, respectively.

Whole-genome sequencing is currently one of the most important methods for analysing phage structure, evolution, and interaction with host bacteria. WGS analysis revealed that the phage XAM237 was a virulent phage and did not contain any antimicrobial resistance, virulence, or lysogeny-associated genes. In addition, genome sequencing revealed that XAM237 carried multiple ORFs for tail fibre proteins (ORFs 131, 134, 135, 168, 169 and 219) that are involved in recognizing host bacteria. The presence of multiple tail fibre proteins may have allowed phage XAM237 to infect multiple strains of pathogenic *E. coli*. Furthermore, the phage XAM237 genome encodes many genes encoding lytic proteins, such as lysozyme and holin, which further confirmed that XAM237 is a virulent phage [37].

In the past 10 years, the application of phages in the control of bacterial diseases in animal reproduction has been widely reported [38–40]. In vitro, phage XAM237 can effectively lyse host cells at various MOIs, even at MOIs as low as 0.01. In a mouse model of the aEPEC strain E711, the phage XAM237 could be maintained at concentrations of at least 10^5^ PFUs/g in the spleen, kidneys and small intestine for up to 18 h after application. In addition, the bacteriological loads in the small intestine, spleen, and kidney of the mice treated with phage were lower than those in the untreated group. These results suggest that phage XAM237 can not only kill target bacteria in vitro but also exert good inhibitory effects in a mouse model.

Finally, phage XAM237 exhibited a broad host spectrum against pathogenic *E. coli*. Furthermore, it can clear bacteria in animals within a short period without side effects and has potential value in the treatment of diseases caused by its target bacteria.

## Data Availability

The complete genome sequence of phage XAM237 has been deposited in the figshare database for reference [41].
